# Efficacy of local neem extracts for sustainable malaria vector control in an African village

**DOI:** 10.1186/1475-2875-7-138

**Published:** 2008-07-23

**Authors:** Rebecca L Gianotti, Arne Bomblies, Mustafa Dafalla, Ibrahim Issa-Arzika, Jean-Bernard Duchemin, Elfatih AB Eltahir

**Affiliations:** 1 Department of Civil and Environmental Engineering, Massachusetts Institute of Technology, 77 Massachusetts Avenue, Cambridge, MA 02139, USA; 2 Centre de Recherche Médicale et Sanitaire, Réseau International des Instituts Pasteur, BP 10877, Niamey, Niger

## Abstract

**Background:**

Larval control of malaria vectors has been historically successful in reducing malaria transmission, but largely fell out of favour with the introduction of synthetic insecticides and bed nets. However, an integrated approach to malaria control, including larval control methods, continues to be the best chance for success, in view of insecticide resistance, the behavioural adaptation of the vectors to changing environments and the difficulties of reaching the poorest populations most at risk,. Laboratory studies investigating the effects of neem seed (*Azadirachta indica*) extracts on *Anopheles *larvae have shown high rates of larval mortality and reductions in adult longevity, as well as low potential for resistance development.

**Methods:**

This paper describes a method whereby seeds of the neem tree can be used to reduce adult *Anopheles gambiae s.l. *abundance in a way that is low cost and can be implemented by residents of rural villages in western Niger. The study was conducted in Banizoumbou village, western Niger. Neem seeds were collected from around the village. Dried seeds were ground into a coarse powder, which was then sprinkled onto known *Anopheles *larvae breeding habitats twice weekly during the rainy season 2007. Adult mosquitoes were captured on a weekly basis in the village and captures compared to those from 2005 and 2006 over the same period. Adult mosquitoes were also captured in a nearby village, Zindarou, as a control data set and compared to those from Banizoumbou.

**Results:**

It was found that twice-weekly applications of the powder to known breeding habitats of *Anopheles *larvae in 2007 resulted in 49% fewer adult female *Anopheles gambiae s.l. *mosquitoes in Banizoumbou, compared with previous captures under similar environmental conditions and with similar habitat characteristics in 2005 and 2006. The productivity of the system in 2007 was found to be suppressed compared to the mean behaviour of 2005 and 2006 in Banizoumbou, whereas no change was found in Zindarou.

**Conclusion:**

With a high abundance of neem plants in many villages in this area, the results of this study suggest that larval control using neem seed powder offers a sustainable additional tool for malaria vector control in the Sahel region of Niger.

## Background

Malaria continues to place a large social and economic burden on African communities. Programs to control malaria transmission typically target the adult primary vectors, using techniques such as bed nets and indoor residual spraying that have a high impact on vectorial capacity. However, these methods are vulnerable to development of vector resistance to insecticides [1-4], vector behavioural adaptation, such as changing preferences for feeding and resting outdoors [5], and logistics and funding problems in reaching the poor, who are most at risk [6]. Historically, environmental management methods that targeted the larval stages of malaria vectors were effective in substantially reducing malaria transmission [7-9]. These methods fell out of favour with the widespread introduction of synthetic insecticides and bed nets, which reduce biting rates and are not dependent on such site-specific knowledge as is required for larval control methods [10,11]. Integrated vector management programmes, employing a variety of tools including larval control, may provide the greatest chance for success in reducing malaria transmission rates [12]. Methods that target the larval stages of mosquitoes have the potential to be effective, low-cost and with low environmental impact [5,13-16]. If modern-day larval control is to be a useful addition to the toolbox of malaria abatement methods, it will need to be both low-cost and sustainable.

**Figure 1:**
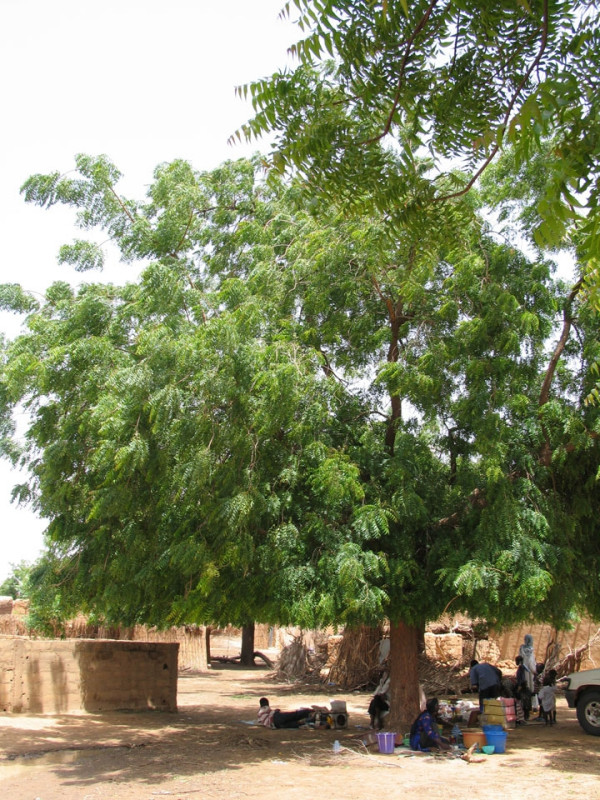
**Neem tree in Banizoumbou village**. A neem tree in the centre of Banizoumbou, adjacent to the village mosque and a shallow groundwater well (the primary drinking water source), provides a convenient place for residents to rest in the shade. Approximately 85 neem trees are present in and immediately surrounding the village.

Neem trees, like that shown in Figure 1, are widespread across the Sahel region of West Africa and are very adaptable and hardy plants [17]. The neem seed kernels contain insecticidal properties due to a combination of approximately 99 active compounds, the most potent of which is azadirachtin, present in the seeds at a concentration of about 5 mg/g of kernel [17]. Neem seed extracts provide a potential larval control method that could be complementary to other malaria abatement methods.

Extracts from neem seeds have documented effects on a variety of insects, which include repellence and anti-feeding, deterrence of egg-laying, inhibition of metamorphosis and disruption of growth and reproduction [17-19]. The extracts are also toxic to crustaceans, particularly aquatic crustaceans, some species of fish, such as gambusia and tilapia, as well as nematodes and snails [17,19] and for these reasons it is generally recommended that neem seed extracts not be used in complex aquatic ecosystems [19]. Although birds and bats are often observed eating the neem fruit in eastern Africa without ill effects, trials have shown that the seed kernel can be toxic to birds if consumed [17]. Neem seed extracts have also shown to be toxic to guinea pigs, rabbits and rats [19] and to produce ill effects in dogs, sheep, goats and calves [17] and thus domestic animals should be prevented from eating stored seeds. Tests have shown that neem seed extracts are non-toxic to beneficial species such as spiders, bees, crickets, many bugs and beetles, and are actually beneficial to earthworms [17].

Although there are several commercial neem-based pesticides available, none are currently used in mosquito control programmes [20]. In recent years, there have been a number of studies conducted to investigate the particular effects of neem extracts on malaria-transmitting mosquitoes. Exposure of anopheline larvae to undiluted neem oil has resulted in 100% mortality within 12 hours [21]. When applied to artificial water bodies every two weeks over a period of three months, emulsified neem oil has been shown to have the same effect on larval mortality and adult density as commonly used synthetic insecticides [22]. A study using a neem oil formulation on third and fourth stage *Anopheles gambiae s.s. *larvae showed 50% inhibition of adult emergence at a concentration of 6 ppm [20]. A study using emulsified neem oil showed that within a three months period (five generations), anopheline larvae failed to develop resistance or change their susceptibility to the oil [22]. Research is ongoing into the potential for neem extracts to provide antimalarial treatments as well as prevention [23-26].

Resistance to neem-based compounds is more likely to develop using a refined larvicide based on a single active ingredient, such as azadirachtin, than if the whole seed is used with its multitude of compounds [20,27]. Because the efficacy of neem is targeted towards the larval stages, it does not have a "knock-down" effect on adult mosquitoes. It is, therefore, thought to be most effective if used to prevent adult mosquito populations from reaching large numbers and will not be as effective in reducing numbers of adult mosquitoes once populations are allowed to establish [19].

The most effective way to use neem is to apply seed extract to breeding sites when population numbers are low, during the dry season, in order to eradicate as many immature mosquitoes as possible and reduce the population available for breeding when conditions become more favourable. Once the rainy season commences, regular applications of seed extract should continue to prevent immature mosquitoes from emerging as adults. The efficacy of neem seed extracts has been shown to degrade under exposure to sunlight within seven days [18,28] and thus the toxicity is non-persistent in the environment. This provides environmental benefits but also means that regular applications of neem seed extracts would be required to maintain efficacy.

Neem trees are abundant in Banizoumbou village, the site of this study in western Niger. The fruits that fall from the trees are left to decompose on the ground where they fall or are dispersed by wind or animals. Hence, the use of neem seeds as an insecticide in this village does not represent the introduction of a new compound to this area. The difference is the location where azadirachtin will be concentrated, from bare ground beneath trees to the pools that provide mosquito habitat, and thus the shift in risk from exposure to neem in this new location. However, these breeding habitats are ephemeral, being temporarily formed during the rainy season in topographic low points, and do not represent complex aquatic ecosystems. The main risk would appear to be to the cattle in the village, if they were to drink from a pool where neem seeds were applied. To avoid this risk, neem seed powder was not applied to the one permanent pool in the village, which is used for cattle watering.

In a short field trial conducted in Mali, neem seed powder was applied on known *Anopheles gambiae s.l.* breeding sites on a single occasion at the end of the dry season, prior to the commencement of rains [29]. This was reported to lead to an 86% reduction in adult female mosquitoes in the trial village obtained during one subsequent indoor spray catch, while the control village saw sustained numbers of adult mosquitoes [29]. While this trial sounds promising, it was too limited to draw conclusions from and the detailed methodology and results remain unpublished. This study presents the first field trial of neem seed extracts produced locally and applied at the village scale over the length of a malaria transmission season.

## Methods

**Figure 2:**
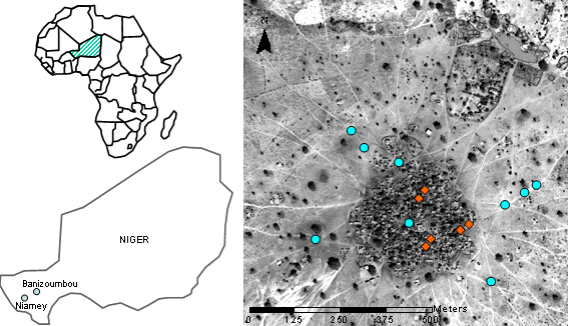
**Locations of Banizoumbou, deployment of light traps and applications of neem seed powder**. The left-hand side of the figure shows the location of Banizoumbou within western Niger, approximately 60 km northeast of Niamey. The right-hand side of the figure shows the locations where the CDC miniature light traps were deployed, indicated by the orange diamonds, and where neem seed powder was applied to ephemeral pools, indicated by the blue circles. The digital photograph is a Quickbird image taken in January 2003, during the dry season. The right hand panel includes material^© ^2003 DigitalGlobe, Inc. ALL RIGHTS RESERVED.

This study was undertaken in Banizoumbou village, located in western Niger, approximately 60 km northeast of Niamey (Figure 2) and home to approximately 1,000 people. The area around Banizoumbou has a typical Sahelian semi-arid landscape, gently sloping topography and savannah vegetation. Neem trees are abundant in the village, with approximately 85 trees within a 500 m radius, but the fruits are not utilised by the residents. This density of neem trees within the village was observed to be typical of villages in the area. The fruiting season is roughly June to August annually. During a rainy season that extends from May to early October and peaks in August, many ephemeral pools form within and around the village in topographic low points, which is a typical feature of the hydrology in this region [30]. These pools do not form complex aquatic ecosystems and were not observed to be utilized by the residents. However, they do provide an ideal breeding habitat for *Anopheles gambiae s.l.*mosquitoes, the major local malaria vector. These pools were the targeted areas for neem seed powder applications. There is only one permanent pool of surface water in the village; it is used primarily for cattle watering and is not used by the people.

Environmental variables, including precipitation, temperature, relative humidity and wind speed and direction, and mosquito abundance have been measured in Banizoumbou since June 2005. Two years' monitoring of environmental conditions and vector dynamics, during 2005 and 2006, enabled a targeted strategy to be developed for the neem seed powder trial in the third year of observations in 2007. The ephemeral pools that form within and adjacent to Banizoumbou were monitored during 2005 and 2006 to determine which pools became habitat for anopheline larvae and it was these pools that were targeted in 2007 for the neem seed powder applications.

Neem seed powder was prepared and applied following suggestions by Schmutterer [17] and a published laboratory trial [31]. Residents of Banizoumbou were asked to collect neem seeds on five occasions throughout the rainy season, beginning in early July when the first ephemeral pools began to appear. The residents were familiar with the seeds and where to find them and were able to easily and quickly collect an adequate supply. Due to the abundance of neem trees within the village, seeds could be gathered mostly from fallen fruit and trees did not have to be stripped of unripe fruit. The fleshy pulp was removed from the outside of the seed casing and seeds were stored either as the bare seed kernel or with the white protective casing around the kernel left intact. Seeds were spread out on grass mats inside a mud brick house to dry for approximately 5–7 days before use.

On the morning of an application day, seeds were crushed into a coarse powder using a mortar and pestle. The mortar and pestle was identical to those used by women in the village for grinding millet and was purchased from a local market in Niamey, to avoid appropriating a mortar and pestle currently used for food preparation. The grinding was carried out by a female resident of the village, using the same technique as is employed for grinding millet. This methodology required only minimal tools – a grass mat for drying seeds, a mortar and pestle for grinding the dried seeds and a bucket for carrying around the powder – that can typically be found within the village. The methodology was designed to be implementable by the village residents and to be low cost.

The first application of neem seed powder occurred on 9^th ^July 2007. At that time, only one ephemeral pool was present in the village. After this initial application, the pool dried out and there was no rain for several days. The next application occurred on 20^th ^July 2007, after rain had created some pools in the village, and thereafter applications continued twice weekly until early October 2007, when all pools dried out completely following cessation of rains. This application frequency was chosen to ensure continued efficacy of the powder, due to the short active lifetime of azadirachtin [18,28].

On each application day, powder was applied to all ephemeral pools in and immediately surrounding the village that were known to be breeding sites for *An. gambiae s.l. *(Figure 2). These ephemeral pools were not observed to establish complex aquatic systems and are not used by the village residents. The powder was carried around in a bucket and liberally sprinkled over the surface of a known breeding pool. The average rate of powder application was approximately 10 g/m^2 ^of pool surface area, with particular attention paid to pool edges where larvae were observed to congregate. No powder was applied to the one permanent pond in the village because of its use by cattle for drinking water, to avoid any toxicity risk. Applications of the neem seed powder in this study were carried out by the authors to ensure consistency of application rates and locations.

Mature neem trees are reported to produce approximately 20 kg of fruit per year, of which the seed kernel accounts for 10% of the weight [31]. Therefore, the 85 neem trees in Banizoumbou are estimated to produce a total of approximately 170 kg of seed kernel per year, during a fruiting season that coincides with the rainy season and thus presence of ephemeral breeding habitats. At an application rate of 10 g/m^2 ^of pool surface area, with twice weekly applications for about 12 weeks, the trees in Banizoumbou could cover a total surface area of about 700 m^2 ^per application. The ephemeral breeding pools in Banizoumbou ranged in size from about 4 m^2 ^to about 200 m^2 ^and the quantity of seeds available was sufficient to adequately cover these pools.

The targeted pools were monitored twice weekly in 2007. Observations were made of pool presence, as an indicator of habitat availability. Adult mosquito populations were monitored using CDC miniature light traps deployed at six locations within the village (Figure 2) in 2005, 2006 and the intervention year 2007. Light traps were deployed weekly from June to November and monthly during the dry season of December to May, commencing in late June 2005 and continuing until November 2007. Captured mosquitoes were identified at a laboratory of Centre de Recherche Médicale et Sanitaire in Niamey, Niger. Records of *Aedes aegypti *and *Culex sp. *mosquito captures were kept along with *An. gambiae s.l. *for comparison. Rainfall was measured at hourly intervals from May 2005 with a tipping bucket rain gauge. Temperature and relative humidity were recorded at 15-minute intervals from August 2005 with a Campbell Scientific CR10 datalogger fitted with a temperature and relative humidity probe.

Data on adult mosquito populations were also collected in the village Zindarou, located approximately 25 km east-southeast from Banizoumbou, to provide a control for the observed behaviour of the adult *An. gambiae s.l.* populations. Zindarou is home to approximately 500 residents and experiences a similar climate to Banizoumbou. CDC miniature light traps were deployed at six locations within Zindarou in 2005, 2006 and 2007 to monitor adult mosquito populations. Deployment was weekly from June to November of each year and monthly during the dry season of December to May, with the last captures being in November 2007. Captured mosquitoes were identified to species at a laboratory of Centre de Recherche Médicale et Sanitaire in Niamey, Niger.

Statistical testing was performed to determine if the relationship between rainfall and anopheline mosquito captures was significantly different in the intervention and non-intervention years. The analysis was performed for both Banizoumbou and Zindarou. It was considered that cumulative rainfall over each season was more appropriate for comparison than hourly or weekly rainfall, because of the non-linear way that breeding pool formation and persistence depends on the rainfall history. At the time of each mosquito capture event (CDC miniature light trap deployment), taken to be midday on the day that the deployed traps were collected, the cumulative rainfall in each year up to that time was calculated. This cumulative rainfall (given in mm) represents the integral of the hourly rainfall from June of each year until the mosquito capture event. Cumulative anopheline mosquito captures were calculated in the same way for each year and in each village.

The paired data points of cumulative mosquito captures and cumulative rainfall were ranked in order of increasing cumulative rainfall for each of years 2005, 2006 and 2007 for each village. For each year, the data points were divided into four bins of equal sample size. In both villages, 12 data points were in each bin for the non-intervention years of 2005 and 2006, and six points were in each bin for the intervention year of 2007. The binned data were combined for 2005 and 2006, to allow comparison of non-intervention years with the intervention year of 2007 alone. The mean and standard deviation of both the cumulative rainfall and cumulative mosquito captures were calculated within each bin for each year. The data points were binned to allow calculation of confidence intervals for these means. 95% confidence limits were calculated for both cumulative rainfall and cumulative mosquito captures with a t-distribution to test the difference of sample means of unknown variance.

Prior to undertaking the field study, preliminary laboratory testing was performed on *An. gambiae s.l. *larvae obtained from a pool in Niamey. This testing confirmed the efficacy of local neem seeds against anopheline larvae.

## Results

### Anopheline mosquito captures

**Figure 3:**
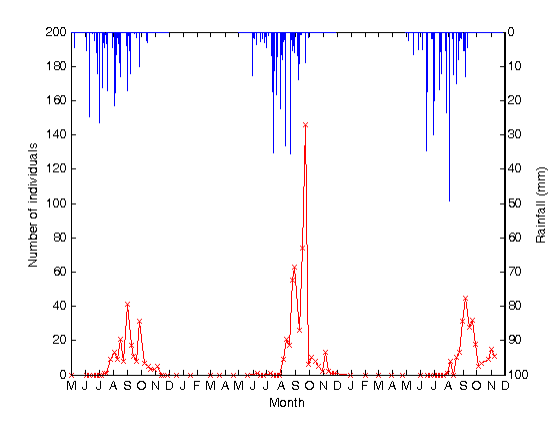
***Anopheles gambiae s.l. *adult female captures and rainfall for rainy seasons 2005, 2006 and 2007**. The figure shows the sum of adult female *An. gambiae s.l. *captures from all six CDC miniature light traps and rainfall observations, for the rainy seasons of 2005 to 2007. Rainfall observations commenced in May 2005 and mosquito sampling commenced in late June 2005. During the months of June to November, when mosquito population abundance increases, sampling was undertaken on a weekly basis. During the dry season months of December to May, sampling was conducted monthly. Mosquito captures are shown in red and recorded on the left-hand axis, with crosses to mark the dates of trap deployments. Hourly rainfall measurements are shown in blue and recorded on the right-hand axis.

Adult female *An. gambiae s.l. *mosquitoes (Figure 3) in Banizoumbou were first captured in 2007 at the end of July, approximately two weeks later than in 2005 and 2006. In all three years, weekly captures increased during July and August from initial weekly captures of single individuals to tens of individuals. Seasonal maximum weekly captures were recorded in late August in 2005 (41 individuals) and in September in 2006 (146 individuals) and 2007 (45 individuals). Weekly captures decreased rapidly to 10 or fewer individuals during late September and early October in all three years. Weekly captures of adult female *An. gambiae s.l. *mosquitoes (Figure 3) during July to September 2007 were generally lower than captures during that same period in 2006, although captures during October and November were comparable in 2006 and 2007. Weekly captures were of similar magnitude throughout the season during 2005 and 2007.

**Figure 4:**
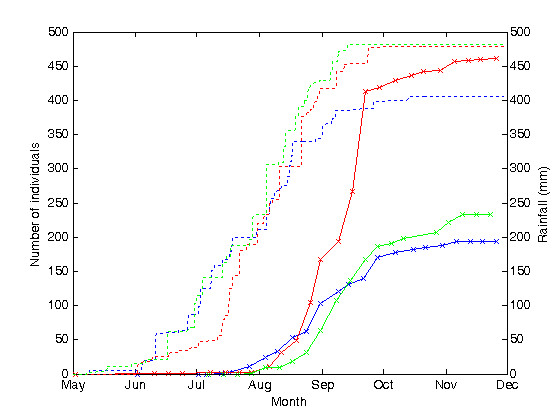
**Cumulative *Anopheles gambiae s.l. *adult female captures and rainfall during rainy seasons of 2005, 2006 and 2007**. Cumulative adult female *An. gambiae s.l. *captures from the sum of all six CDC miniature light traps are shown by the solid lines, with crosses to mark the dates of each trap deployment, and the numbers captured are recorded along the left-hand axis. Cumulative rainfall is shown by the dashed lines, updated hourly, and is recorded along the right-hand axis. The cumulative data represent the integral over time of the data shown in Figure 3. For both data sets, 2005 data is in blue, 2006 data is in red and 2007 data is in green.

Figure 4 shows the cumulative captures of adult female *An. gambiae s.l. *mosquitoes in each year. Cumulative captures over 2007 (233 individuals) were 49% less than the cumulative captures over 2006 (460 individuals) and 20% greater than cumulative captures over 2005 (193 individuals). Figure 4 shows that more than half of the total cumulative captures in 2006 occurred during September, with weekly captures increasing rapidly during this time. However, in 2005 and 2007, weekly captures remained consistent throughout the season, such that cumulative captures increased at a moderate rate and did not accelerate during the later part of the season as was observed in 2006. Figure 4 also shows that increases in cumulative captures in 2007 were delayed relative to 2006 by about 2 weeks, despite the first rains occurring about two weeks earlier in 2007 than in 2006.

### Environmental variables

Environmental variables of rainfall, air temperature and relative humidity were recorded during the non-intervention and intervention years to determine if ambient conditions could have contributed to changes in observed adult mosquito captures.

Figures 3 and 4 show that rain began in Banizoumbou in early- to mid-May in both 2005 and 2007. Rainfall in the early part of the season was similar in both 2005 and 2007. However, there was more rainfall recorded towards the end of the season in 2007 than in 2005, such that total cumulative rainfall in Banizoumbou was 482 mm in 2007, approximately 19% greater than the 405.5 mm measured in 2005.

Figures 3 and 4 show that rain began in Banizoumbou in early June in 2006. Cumulative rainfall measured in 2006 was 478.3 mm, comparable to the 482 mm measured in 2007. Figures 3 and 4 also show that more rainfall was earlier in the season (May to June) in 2007 relative to 2006, whereas 2006 experienced more rainfall in the later part of the season (August to September) than 2007.

**Figure 5:**
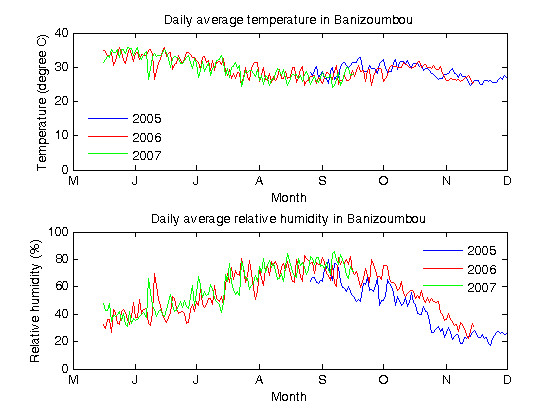
**Temperature and relative humidity in Banizoumbou during rainy seasons of 2005, 2006 and 2007**. The figure shows temperature (top panel) and relative humidity (bottom panel) measurements taken at 15-minute intervals for the rainy seasons of 2005, 2006 and 2007. Sampling began in August 2005 and was continuous until mid-November 2006, when a technical fault led to cessation of data collection for that year. Data was received up until mid-September in 2007, after which a technical fault caused observations to be recorded only during daylight hours. Hence daily average values have not been presented after this time. In both panels, 2005 data is in blue, 2006 data is in red and 2007 data is in green.

Figure 5 shows daily mean air temperatures (top panel) and daily mean relative humidity values (bottom panel) in Banizoumbou. The data indicate that ambient air temperature and relative humidity observations were similar in 2007 compared with both 2005 and 2006. The average daily mean air temperatures were 29°C in 2005 (standard deviation of 2°C), 30°C in 2006 (standard deviation of 3°C) and 30°C in 2007 (standard deviation of 3°C). Temperatures were higher in the early part of the season (32–34°C in May to June) than in the later part of the season (27–29°C in August to September), as the onset of regular rain events had a cooling effect.

The average daily mean relative humidity values were 44% in 2005 (standard deviation of 18%), 56% in 2006 (standard deviation of 16%) and 59% in 2007 (standard deviation of 15%). Relative humidity values were low in the early part of the season (37–47% in May to June), rose to high levels during the later part of the season when rain events were regular (73–76% in late-July to early-September) and then decreased again after the cessation of rains in November (24–31%).

### Breeding pool availability

Observations of pool persistence were recorded for the ephemeral breeding pools during the non-intervention and intervention years to determine if changes to breeding habitat availability could have contributed to changes in observed adult mosquito captures.

**Figure 6:**
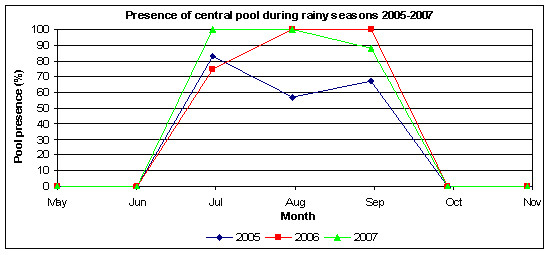
**Presence of central pool during rainy seasons 2005–2007**. The figure shows the proportion of monitoring events in each month during the rainy seasons of 2005 to 2007 during which the central pool was present as an indication of the persistence of this pool throughout each rainy season. 2005 data is shown in blue, 2006 data is shown in red and 2007 data is shown in green.

Figure 6 shows the persistence of the central pool (located in the centre of the village on Figure 2) during the period June to November in 2005, 2006 and 2007, measured as the percentage of monitoring visits in each month during which the pool was present. This pool was observed in each year to contain the highest abundance of larvae throughout the rainy season and thus its persistence is indicative of the availability of breeding habitats throughout the village. Generally, the central pool was absent from October to June and was sporadically present from July to September each year.

Figure 6 shows that the pool was present on more occasions during 2006 and 2007 than in 2005. Averaged over the period July to September, the central pool was present on 69% of monitoring visits in 2005, 92% of monitoring visits in 2006 and 96% of monitoring visits in 2007. The pool was present on more occasions during July in 2007 than in 2006, consistent with the higher rainfall received in the early part of the season in 2007 compared with 2006. The pool was present on fewer occasions during September in 2007 than in 2006, again consistent with the lower rainfall received in the later part of the season in 2007 compared with 2006. Other ephemeral pools shown in Figure 2 were generally less persistent than the central pool, but the relative persistence difference between years was similar to the central pool, with greater persistence during 2006 and 2007 than 2005. Thus habitat availability from the perspective of pool persistence was considered to be greater in 2006 and 2007 than 2005.

### Culicine mosquito captures

Captures of culicine mosquitoes, primarily *Culex sp. *and *Aedes aegypti*, were recorded during non-intervention and intervention years as an indication of any general environmental effects occurring in Banizoumbou that might affect mosquito populations, given that culicine and anopheline mosquitoes share the same ambient environment (air temperature and humidity, rainfall etc) but not the same breeding sites in Banizoumbou.

**Figure 7:**
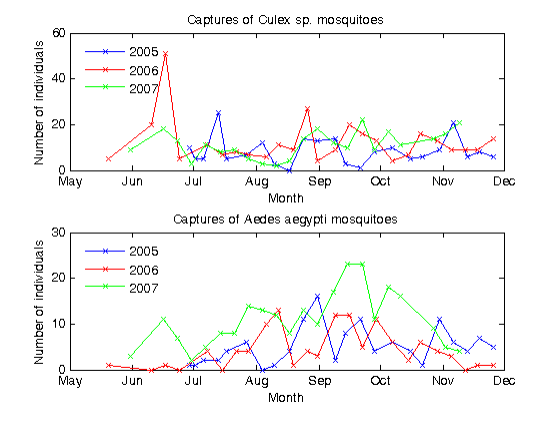
**Captures of *Culex sp. *and *Aedes aegypti *mosquitoes during rainy seasons 2005, 2006 and 2007**. The figure shows the sum of *Culex sp. *(top panel) and *Aedes aegypti *(bottom panel) captures from all six CDC miniature light traps, for the rainy seasons 2005 to 2007. Sampling commenced June 2005. During the months of June to November, when mosquito population abundance increases, sampling was undertaken on a weekly basis. During the dry season months of December to May, sampling was conducted monthly. Crosses mark the dates of trap deployments. In both panels, 2005 data is shown in blue, 2006 data is shown in red and 2007 data is shown in green.

Weekly captures from June to November in 2005, 2006 and 2007 are shown in Figure 7. The top panel shows captures of *Culex sp. *and the bottom panel shows captures of *Ae. aegypti *mosquitoes. The figure shows that weekly captures of *Culex sp. *were relatively consistent throughout each season, with little temporal variation and an average weekly capture of 9 individuals in 2005 (standard deviation of 6), 12 individuals in 2006 (standard deviation of 10) and 11 individuals in 2007 (standard deviation of 6). Weekly captures of *Ae. aegypti *mosquitoes showed more temporal variation, with higher captures recorded during August to October in each season than at other times. Average weekly captures of *Ae. aegypti *were 5 individuals in 2005 (standard deviation of 4), 4 individuals in 2006 (standard deviation of 4) and 11 individuals in 2007 (standard deviation of 6). More *Aedes aegypti *mosquitoes were captured during September in 2007 than in 2005 and 2006, but at other times during each season the captures were comparable between years.

### Relationship between rainfall and anopheline abundance

**Figure 8:**
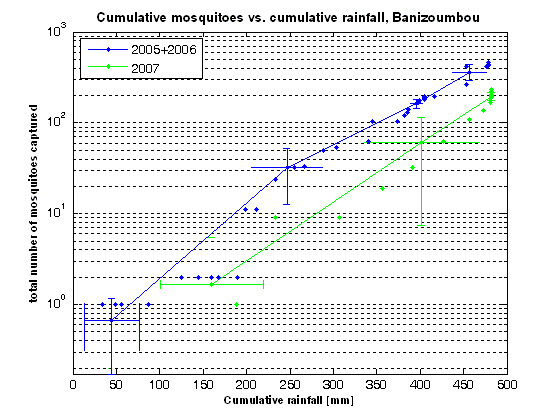
**Correlation between cumulative *Anopheles gambiae s.l. *captures and cumulative rainfall for 2005, 2006 and 2007 in Banizoumbou**. The figure depicts cumulative mosquito captures in Banizoumbou plotted against cumulative rainfall on a log-linear axis. The data points represent the cumulative rainfall, calculated from June of a given year until the time of a mosquito sampling event, and the cumulative anopheline mosquito captures in a given year up to the time of that event. For each year, the data points were divided into four bins of equal sample size. 12 data points were in each bin for the non-intervention years of 2005 and 2006 and 6 data points were in each bin for the intervention year of 2007. The mean and standard deviation of both the cumulative rainfall and cumulative mosquito captures were calculated within each bin for each year. 95% confidence limits were calculated with a t-distribution and are shown as the error bars around each data point. The combined 2005 and 2006 data is shown in blue and 2007 data is shown in green.

**Figure 9:**
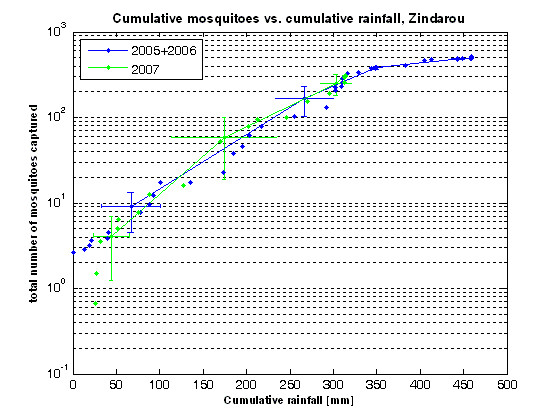
**Correlation between cumulative *Anopheles gambiae s.l. *captures and cumulative rainfall for 2005, 2006 and 2007 in Zindarou**. The figure depicts cumulative mosquito captures in Zindarou plotted against cumulative rainfall on a log-linear axis. The data points represent the cumulative rainfall, calculated from June of a given year until the time of a mosquito sampling event, and the cumulative anopheline mosquito captures in a given year up to the time of that event. For each year, the data points were divided into four bins of equal sample size. 12 data points were in each bin for the non-intervention years of 2005 and 2006 and 6 data points were in each bin for the intervention year of 2007. The mean and standard deviation of both the cumulative rainfall and cumulative mosquito captures were calculated within each bin for each year. 95% confidence limits were calculated with a t-distribution and are shown as the error bars around each data point. The combined 2005 and 2006 data is shown in blue and 2007 data is shown in green.

Figures 8 and 9 show the relationship between cumulative rainfall and cumulative *An. gambiae s.l. *captures for the intervention and non-intervention years in Banizoumbou and Zindarou respectively. The figures depict paired data points of cumulative mosquito captures and cumulative rainfall, which have been ranked in order of increasing cumulative rainfall for each of years 2005, 2006 and 2007 in each village and then divided into four bins of equal sample size. Figures 8 and 9 show the means and 95% confidence limits for each bin, with the data points left on the plots. The binned data were combined for 2005 and 2006, to allow comparison of non-intervention years with the intervention year of 2007 alone. The figures are plotted on log-linear axes.

Figure 8 shows that the productivity of the system in 2007 in Banizoumbou was significantly below the range of mean behaviour observed in 2005 and 2006. In contrast, in Figure 9 the lack of deviation of mosquito captures in 2007 from 2005 and 2006 shows that in Zindarou the productivity of the system in 2007 was within the range of mean behaviour observed in 2005 and 2006. This empirical evidence is consistent with the hypothesis that neem had a significant impact at the village scale in Banizoumbou.

## Discussion

The relationship between rainfall and *An. gambiae s.l. *abundance in Banizoumbou is highly non-linear, as shown in Figure 8. The figure is depicted on a log-linear axis, so the apparently linear nature of the relationship presented on a logarithmic scale is actually very non-linear. As is shown by the deviation of mosquito captures in 2007 from the mean behaviour observed in 2005 and 2006, there was a change in the productivity of the system in Banizoumbou in 2007 that was not observed in the control village Zindarou. This indicates that *An. gambiae s.l. *populations were significantly suppressed in Banizoumbou in 2007.

There are many factors that could potentially influence the populations of *An. gambiae s.l. *mosquitoes in Banizoumbou besides the application of neem seed powder to breeding habitats. The collection of data related to ambient environment, breeding pool availability and culicine mosquitoes was undertaken to determine if these other factors could have affected anopheline mosquito abundance.

The results show that environmental variables of rainfall, temperature and relative humidity were comparable between the non-intervention and intervention years. Rainfall was greater in 2007 compared with 2005, but similar in 2006 and 2007. Given that ambient environmental variables were so similar in 2006 and 2007, it is suggested that these factors should not have significantly affected anopheline mosquito populations in 2007 relative to 2006.

Observations of breeding habitat availability, as indicated by the presence of the pool shown in Figure 6, indicate that availability was greater in 2006 and 2007 than 2005. Habitat availability was comparable in 2006 and 2007 and this should. therefore, not be a significant factor affecting *Anopheles *abundance in 2007 relative to 2006.

The stability of culicine mosquito populations, during a time when the anopheline mosquito populations were significantly altered, indicates that there was an impact on the mosquito life cycle that only affected the anopheline species. It was observed in each of the study years that, in Banizoumbou, culicine mosquitoes tend to breed in different habitat locations than *An. gambiae s.l. *and would not, therefore. have been affected by the neem applications. However, all mosquitoes share the same ambient environment, in terms of temperature, humidity, wind speed and prevailing direction, and populations of human inhabitants. Although the behaviour and tolerance to dryness are different for *Ae. aegypti*, *Culex sp. *and *An. gambiae s.l.*, the observed population stability of the culicine mosquitoes over the three years shows that there was no major climatic effect on mosquito populations in 2007. Thus, the different behaviour of *An. gambiae s.l. *in 2007 can be attributed to the fact that anopheline mosquitoes were affected in their breeding habitat. Given that anopheline breeding habitat characteristics were similar between 2006 and 2007, it is suggested that the observed difference in *An. gambiae s.l. *abundance in 2007 compared with 2006 is due to the addition of neem seed powder applications.

A comparison of the powder's efficacy with previous studies is difficult as they have been conducted under laboratory or highly controlled field conditions, generally using concentrated neem extracts. The method presented here used the entire seed and the powder was produced using minimal tools. Field effects such as wind dispersal, dissolution and mechanical mixing from birds and carts would have reduced the impact of the applied powder. It is, therefore, considered that the neem seed powder performed favourably under true field conditions in this study.

A previous laboratory study has recorded approximately 25% reduction in longevity in adults that were exposed to a neem oil formulation at a concentration of 4 ppm as larvae [20]. This effect is important as a reduction in average adult daily survival rate is crucial for lowering a vector's disease transmission potential. Although adult longevity was not measured in this study, it is possible that adult longevity was also affected by the applications of neem seed powder and contributed to the observed reductions in *An. gambiae s.l. *abundance in 2007.

Accurate, quantitative data on *An. gambiae s.l. *larval presence in the breeding pools were not collected with sufficient sampling density in space and time to provide quantitative measures of larval abundance. However, it is known that neem seed extracts affect mosquito larvae primarily by inhibiting metamorphosis and suppressing adult emergence [17]. It is conceivable that a breeding habitat where neem has been applied could exhibit a comparable larval abundance to an unaffected breeding habitat. However, the neem-affected habitat would not be expected to produce as many adult mosquitoes as the unaffected habitat. Hence monitoring of larval abundance may not capture the impact of neem seed powder on mosquito populations. For these reasons, it is suggested that monitoring of adult populations is more appropriate in this study for assessing the effectiveness of neem in a field setting than larval abundance.

The techniques used in this study for seed preparation and application could easily be taught to residents and carried out in other villages. The main obstacle to this technique being implemented by residents in other locations is the ability to identify *Anopheles *breeding habitats and thus to appropriately target the applications. This would be particularly important if many pools were present in a village and neem seeds were not sufficient to cover every surface water body. In those cases especially, targeting of powder only to pools that were known to be breeding habitats would be important for efficient use of time and resources. As part of this study, residents of Banizoumbou were educated about the mosquito life cycle, the connection between malaria illness and the ephemeral breeding pools, and the reasons for applying powder to these pools. This kind of education, as well as some training in habitat identification, would be necessary to implement this technique in other locations.

The most significant cost of this method is the labour and time required for collection of seeds, preparation of the powder and application to pools. It is estimated that in Banizoumbou, these tasks would require three days per week of labour for one person throughout the transmission season, which lasts about 16 weeks in this region. Using estimates of local daily labour wages, it is anticipated that this intervention would cost about US$200 per year, or roughly US$0.20 per person per year for each Banizoumbou resident.

## Conclusion

Given the comparability between 2006 and 2007 of all the datasets described above, a similar *An. gambiae s.l. *abundance could have been expected in 2007 as in 2006. Similarly, the observed differences between 2005 and 2006 would have been expected to repeat in 2007. However, the data shows that in Banizoumbou 2007 did not behave as expected and *An. gambiae s.l. *populations were suppressed relative to expectations. The only significant change made in 2007 was the application of neem seed powder to *An. gambiae s.l. *habitats and, therefore, it is suggested that the observed difference in *An. gambiae s.l. *abundance in 2007 can be attributed to the neem seed powder.

The results of this study suggest that neem seeds could provide an appropriate, sustainable larvicide for the malaria vector *An. gambiae s.l. *in the Sahel region of Niger and adjacent areas having similar environmental characteristics and vector dynamics. A larger-scale study is recommended to test the efficacy of this method in other, similar villages in the region. A multi-year trial is also recommended to test for any long-term residual effects of using the powder. Although this method will not replace other forms of malaria abatement in Africa, it is suggested that neem seed powder could be a useful additional tool in the fight against this infection.

## Authors' contributions

RLG participated in the field trial and drafted the manuscript. AB led the field trial, conducted the statistical analysis and participated in writing the final version of the manuscript. MD participated in the field trial and conducted the laboratory test. II–A participated in the field trial. J–BD provided in-field supervision, advice for study design and coordination and participated in writing the final version of the manuscript. EABE provided advice for study design, remote supervision and participated in writing the final version of the manuscript. All authors read and approved the final manuscript.
